# Psychological Frailty in Later Life: A Critical Narrative Review of Integrative Interventions and the Proposed RESERVE-7 Heuristic

**DOI:** 10.3390/healthcare14203423

**Published:** 2026-10-13

**Authors:** Fatma Magdi Ibrahim, Ghada Shahrour, Khaled Elbarbary

**Affiliations:** 1 Faculty of Nursing, Mansoura University, Mansoura 35516, Egypt; 2 RAK College of Nursing, RAK Medical and Health Sciences University, Ras Al Khaimah P.O. Box 11172, United Arab Emirates; 3 Faculty of Nursing, Jordan University of Science and Technology, Irbid 22110, Jordan; 4 Faculty of Medicine, Mansoura University, Mansoura 35516, Egypt; khaledelbarbary7766@gmail.com

**Keywords:** psychological frailty, geropsychology, psychological reserve, integrative care, aging, psychotherapy, mindfulness, lifestyle intervention, social connection, person-centered care

## Abstract

Background: Psychological frailty may describe reduced capacity to adapt to stress in later life, but definitions vary and its added clinical value is unknown. Methods: We conducted a critical narrative review of MEDLINE via PubMed (1 January 2015 to 29 August 2026), citation chains, and selected guidance. The original core query returned 114 records; the focused reconciliation added construct-specific reports. Forty-nine sources inform this synthesis. Results: Trials have measured psychological-frailty domains, and D-SCOPE permitted a psychological-domain threshold as one of several enrollment routes. Freitag and Schmidt reported small short-term improvements in frailty and mental health; D-SCOPE reported no significant six-month within-group change in psychological frailty; and MIND-STEP reported a psychological-frailty risk difference of −13.87 percentage points at 12 weeks (95% CI −25.04 to −2.69). These measures are not interchangeable with our proposed affective–adaptive definition. We found no trial that exclusively recruited participants using a validated version of that proposed construct. Adjacent evidence concerns defined symptoms and functional outcomes. We propose RESERVE-7 as an unvalidated heuristic for identifying goals, reversible drivers, an initial feasible intervention, treatment burden, and follow-up. Conclusions: Construct-specific intervention findings deserve explicit consideration, but they do not validate psychological reserve or RESERVE-7. Prospective validation is needed before adoption.

## 1. Introduction

Older adults are a heterogeneous population, and aging itself is not a mental-health problem. Many people retain high levels of psychological well-being, autonomy, and social contribution across later life. Some, however, experience interacting stressors such as bereavement, multimorbidity, sensory impairment, pain, discrimination, economic strain, or reduced access to supportive relationships. These exposures are neither universal nor intrinsic to age. The World Health Organization (WHO) estimates that approximately 14.7% of adults aged 70 years or older live with a mental disorder and emphasizes that late-life mental-health conditions are often underrecognized and undertreated [1]. WHO Integrated Care for Older People (ICOPE) guidance recommends coordinated, person-centered assessment of intrinsic capacity, social-care needs, and individual goals [2]. The clinical question is therefore how to respond when distress, reduced agency, cognitive concerns, physical vulnerability, and social disconnection reinforce one another in a particular person, without assuming that all older adults are frail.

Frailty is one clinical language for vulnerability to stressors, not a synonym for old age. Biomedical accounts describe diminished physiological capacity to withstand stressors [3], and the physical phenotype operationalizes frailty through weight loss, exhaustion, weakness, slowness, and low activity [4]. These formulations do not fully address the affective and adaptive processes through which a person interprets threat, mobilizes support, maintains routines, and preserves purpose. Psychological frailty has been proposed to address this gap. Its scientific basis remains unsettled: one scoping review identified 28 instruments across 58 studies, with marked variation in the inclusion of mood, cognition, fatigue, and other variables [5], and another recommended clearer separation of affective and cognitive frailty [6]. Fitten earlier described psychological frailty in terms of reduced cognitive, mood, and motivational resilience under stress [7], while a recent Psychological Frailty Index used accumulated deficits and requires independent replication and evaluation of incremental validity [8].

This conceptual uncertainty matters clinically. If psychological frailty becomes a broad container for sadness, memory complaints, loneliness, and fatigue, it may obscure treatable depression, delirium, neurocognitive disorder, grief, pain, or social deprivation. If the construct is rejected altogether, clinicians may miss a real pattern: a person can remain below diagnostic thresholds yet lose the adaptive capacity needed to recover after hospitalization, bereavement, or functional decline. The Tilburg Frailty Indicator partly recognized this multidimensionality by incorporating cognition, depressive symptoms, anxiety, and coping within a broader frailty assessment [9], whereas the consensus construct of cognitive frailty specifically links physical frailty with cognitive impairment in the absence of dementia [10]. The challenge is therefore not to add another label, but to create a formulation that guides proportionate, humane action.

The lived experience of frailty reinforces this caution. Older adults often understand vulnerability in terms of loss of independence, uncertainty, and disrupted identity; some actively resist the word frailty because it feels reductive or stigmatizing [11]. Frailty is not a person’s identity, nor is it destiny. Longitudinal evidence also suggests movement in both directions: in a cohort of 4033 older adults, improvement in psychological resilience was associated with greater odds of frailty improvement and lower odds of deterioration over three years, although causal inference is not possible [12]. This dynamic view motivates the present review.

This critical narrative review has three aims: (1) to clarify the boundaries and possible clinical utility of psychological frailty; (2) to examine intervention evidence from psychological-frailty research and adjacent populations; and (3) to propose a parsimonious, testable heuristic for selecting and adapting care. The review does not assume that older adults form a frailty cohort or that psychological frailty is an established diagnosis. Its central proposition is narrower: when a specific person experiences interacting and potentially modifiable problems, care should target the most consequential feedback loop while limiting avoidable workload.

## 2. Methods

### 2.1. Review Design and Question

A critical narrative review was selected because the literature spans unstable constructs, intervention classes, outcomes, and study designs that cannot be combined meaningfully in a single pooled estimate. The review was planned and self-audited against the Scale for the Assessment of Narrative Review Articles (SANRA) [13]. SANRA informed reporting quality but was not used as a substitute for systematic-review methods. The synthesis distinguishes study findings from author interpretation and direct evidence from extrapolation, consistent with guidance on transparent narrative reviewing [14].

The guiding question was: among adults aged 60 years or older with psychological or multidomain vulnerability, which intervention components may support adaptive capacity, through what proposed mechanisms, for whom, and at what burden? In this article, psychological reserve is an author-defined formulation device referring to accessible affective, agentic, cognitive, social, and environmental resources under stress. It is not an established latent trait or validated measure. Psychological frailty is treated provisionally as reduced access to such resources, not as a diagnosis.

### 2.2. Search Strategy and Eligibility

The original core construct query returned 114 records; this is the original query yield, not a revised total across the focused reconciliation and module searches. All 114 titles and abstracts were screened. Forty-nine cited sources inform the present synthesis, including five added construct-specific publications. We do not claim that all relevant intervention trials were captured: targeted searches and citation chaining were iterative, without a de-duplicated record register or duplicate independent screening.

Eligible evidence included reviews, randomized intervention reports, major cohorts, consensus papers, and implementation guidance relevant to later-life care. Eligible populations had a reported mean or median age of ≥60 years, included ≥80% of participants aged ≥60 years, or contained an extractable subgroup meeting these criteria. We classify each construct-specific intervention on three independent axes: population directness (psychological frailty alone or as one eligible domain versus no psychological-frailty criterion), outcome directness (a distinct psychological-frailty measure or subscale reported versus only a multidomain total or other outcome), and construct concordance (agreement with our proposed affective–adaptive formulation; none should be presumed concordant solely from a label). Protocols are separated from results reports. Pharmacotherapy-only studies were excluded; dementia-specific studies were used only for transferable access or implementation lessons. English-language records were used. F.M.I. conducted the initial selection, and two authors reviewed the final source set. No review protocol was developed, and no duplicate screening, formal risk-of-bias assessment, or meta-analysis was undertaken.

### 2.3. Synthesis

Evidence was organized by intervention mechanism and, for construct-specific reports, by population directness, outcome directness, and construct concordance. We extracted study design, population, operational definition, sample, numerical result, follow-up, and reporting of attrition, adherence, adverse events, acceptability, and workload where available in the consulted sources. “Not reported” denotes an item not located in the consulted report and does not prove it was never collected. Supplementary Table S4 distinguishes reported limitations from our implementation inferences. No identified trial exclusively recruited participants using a validated affective–adaptive definition corresponding to ours. RESERVE-7 remains an author-derived mnemonic, not a validated pathway or treatment package.

### 2.4. Use of Generative Artificial Intelligence

OpenAI ChatGPT was used to support language editing, structural clarity, consistency checks, and drafting, as disclosed in the Acknowledgments. It was not used to generate or analyze study data. The authors verified the scientific claims, references, interpretation, and final wording and take full responsibility for the manuscript.

## 3. Psychological Frailty: Useful Target or Conceptual Overreach?

### 3.1. Boundaries and a Working Definition

The literature does not justify treating psychological frailty as a discrete disorder or an inevitable consequence of aging. Measures variably include low mood, anxiety, coping, cognition, fatigue, and social factors [5,6]. This creates circularity when an intervention improves the same symptom used to define the construct. It also creates overlap with depressive disorders, cognitive frailty [10], social frailty, resilience, coping flexibility, self-efficacy, intrinsic capacity, and general adaptive capacity. Fitten’s formulation emphasized vulnerability of cognition and mood under stress [7]; more recent deficit-accumulation work has operationalized a Psychological Frailty Index [8]. Neither approach yet demonstrates that psychological frailty adds clinically useful information beyond established constructs.

For the purpose of this review, we propose a working definition rather than a consensus definition: psychological frailty is a context-sensitive and potentially reversible state in which reduced affect regulation, agency, coping flexibility, or access to cognitive, social, and environmental resources may limit adaptation to stress and increase the risk of persistent distress or functional decline. Cognition is treated as a modifier because including objective impairment in the core definition would collapse the construct into cognitive frailty; social adversity and physical vulnerability are treated as contextual drivers because locating deprivation or disability solely within the individual would be conceptually and ethically misleading. These boundaries are provisional and require empirical testing against alternative definitions [5,6,7,8,10]. Table 1 summarizes the boundaries between these constructs.

### 3.2. Psychological Reserve as a Mechanistic Bridge

In this review, reserve is a clinical metaphor for resources that may buffer stress, not an independently validated construct. It differs from resilience, which describes observed adaptation over time; coping flexibility, which concerns adjustment of coping strategies; self-efficacy, which is a belief about capability; cognitive reserve, which concerns tolerance of neuropathology; and intrinsic capacity, which WHO defines across physical and mental domains [2,7,8,12]. Pain, sleep disruption, low confidence, inactivity, sensory loss, and social withdrawal may form reciprocal loops, but these relationships should be treated as hypotheses unless longitudinal mediation has been demonstrated.

The formulation may help explain why similar symptom scores call for different assessments. Withdrawal may be related to hopelessness and avoidance, untreated hearing loss and fear of embarrassment, inaccessible transport after bereavement, or several of these mechanisms. Psychological reserve is therefore used only to organize questions about modifiable mechanisms, retained strengths, and environmental resources while preserving diagnostic assessment. Whether it improves prediction, treatment selection, or outcomes beyond established approaches remains unknown [5,6,8].

### 3.3. A Humanized Formulation

Consider a composite clinical illustration. After her spouse dies, a 79-year-old woman sleeps poorly, skips meals, stops walking to her community center, and increasingly declines invitations because group conversation has become hard to follow. A depression screen is positive. Depression treatment may be appropriate, but a depression-only formulation misses grief, hearing difficulty, disrupted routine, nutrition, mobility, transport, and the loss of a valued role. Equally, calling her psychologically frail without asking what matters to her would merely replace one narrow label with another. A reserve-oriented formulation begins with her priorities—perhaps sleeping, hearing her granddaughter, and returning to choir—and selects interventions that make those goals more attainable.

This example is intentionally non-identifying and is not evidence. Its purpose is to show why integration should be organized around the person’s lived circumstances rather than around service silos. The most humane formulation is also often the most mechanistically precise: it locates distress within biography, body, relationships, and environment, while preserving the possibility of recovery.

## 4. Assessment for Action

Assessment should be proportionate and should change care. A possible two-stage process, offered as a provisional practice consideration, begins with a multidomain screen covering mood and anxiety, recent loss or stress, subjective cognition, sleep, pain, mobility, nutrition, hearing and vision, loneliness and participation, medication burden, daily function, retained strengths, and the person’s own priority. Existing tools may contribute, including symptom scales, frailty measures, cognitive screens, and loneliness measures, but no instrument should be assumed to diagnose psychological frailty [2,5,6,8,9]. A second stage can collaboratively identify feedback loops, protective resources, access barriers, and risks.

Established clinical duties take priority over the proposed heuristic. New confusion or fluctuating attention requires assessment for delirium; suicidal ideation requires direct risk assessment and an appropriate safety response; and suspected abuse, neglect, malnutrition, medication toxicity, severe substance-related harm, or rapidly progressive change requires timely escalation [1,2]. Sensory and communication accommodations should precede conclusions about cognition or engagement. Decision-making capacity is specific to the decision, and caregiver involvement should occur with the older adult’s consent whenever possible.

For prospective testing, a minimum dataset for repeated assessment could include one person-valued goal, one symptom or distress measure, one functional or participation outcome, one established measure of coping or self-efficacy, and one measure of treatment workload or feasibility. Goal attainment and return to a meaningful activity may be important alongside symptom change. This proposed dataset has not been validated for psychological frailty and should support, not replace, clinical conversation [2,15,16].

## 5. Evidence for Integrative Intervention Modules

### 5.1. Psychological Therapies

Studies of psychological therapies provide relatively consistent adjacent evidence when depressive or anxiety symptoms are present; they do not provide direct evidence for psychological frailty. A 2024 meta-analysis of 13 RCTs involving 2079 community-dwelling older adults with subthreshold depression found reduced depressive symptoms immediately after psychological intervention (SMD −0.58) and a smaller effect at follow-up (SMD −0.31); anxiety and quality of life also improved modestly [17]. Problem-solving therapy has evidence for major depression in older adults [18], and low-intensity interventions can reduce late-life depression and anxiety, although delivery formats and comparators vary [19].

Cognitive behavioral therapy (CBT), behavioral activation, and problem-solving therapy may require adaptation for pacing, sensory access, cognition, literacy, and goals. These adaptations should be based on individual need rather than assumptions about age. Behavioral activation can link mood treatment to mobility and social participation; problem-solving can address transport or care-navigation barriers. Restored agency is a plausible mechanism, but mediation has not been established in psychologically frail samples.

A well-established adjacent model is collaborative and stepped care. In the IMPACT trial, 1801 older primary-care patients with depression were randomized to usual care or up to 12 months of collaborative care involving a depression care manager, primary-care and specialist collaboration, education, behavioral activation, medication support when appropriate, and relapse planning [20]. This evidence supports coordinated depression care, not a psychological-frailty pathway. Medication support is included here because it was one component of a multicomponent service; pharmacotherapy-only trials remained outside this review.

### 5.2. Mindfulness and Mind–Body Approaches

Mindfulness-based interventions may improve selected emotional and quality-of-life outcomes in some older adults. A meta-analysis reported benefits of mindfulness-based stress reduction for depression, anxiety, and stress [21], and a later synthesis of 26 studies reported improvements in depression, anxiety, quality of life, and working memory, with uncertainty for other cognitive outcomes [22]. One RCT in older adults with depression reported improvements in depression, emotion regulation, and sleep [23]. These studies did not enroll participants on the basis of psychological frailty, and proposed effects on reserve remain untested.

These findings support mindfulness as an option, not a universal first-line module. Long sessions, extensive home practice, trauma-related distress, severe rumination, cognitive impairment, pain during stillness, and cultural or spiritual mismatch can reduce acceptability. Short, guided practices linked to a concrete goal—settling before sleep, noticing early escalation, or tolerating breathlessness during activity—may impose less burden. Consent requires explaining that mindfulness is a skill practice, not a demand to accept preventable suffering or inadequate care.

### 5.3. Life Review, Reminiscence, and Meaning

Life review and reminiscence may support autobiographical continuity, identity, and meaning. Freitag and Schmidt randomized 198 older adults (mean age 75.1 years) to oral or written biographical disclosure, diary writing, or control, measuring frailty with the Tilburg Frailty Indicator (TFI) at baseline, immediately after the intervention, and at three months [24]. Baseline frailty prevalence was 39.9%; the report described small improvements in frailty and mental health in the oral, structured-writing, and diary groups, with no benefit to physical health. Its abstract does not provide a separate numerical TFI psychological-domain effect, so the result must not be represented as proof that the affective–adaptive construct changed. Two broader syntheses support selected life-review outcomes but contain heterogeneous formats and comparators [25,26].

Meaning-centered work should not romanticize adversity. Its function is to help a person locate choice, connection, and coherence where these remain possible. Structured life review may be combined with behavioral activation, for example, transforming a valued identity as a teacher, gardener, or caregiver into a feasible present-day role. Clinicians should monitor for distress when traumatic memories emerge and adapt methods for cognitive or sensory limitations. The therapeutic endpoint is increased integration, self-compassion, or purposeful action rather than a polished life story.

### 5.4. Exercise and Multidomain Lifestyle Intervention

Physical activity can influence mastery, sleep, social exposure, mobility confidence, and brain health, but the evidence reviewed here arises from cognitive or physical frailty. A 2024 meta-analysis of 11 trials involving 2222 older adults with cognitive frailty found improvements in global cognition, grip strength, lower-limb function, frailty, and depressive symptoms; findings for activities of daily living and quality of life were inconclusive [27]. An RCT also reported improvements in frailty, physical performance, and inflammatory biomarkers [28]. Neither source tested psychological frailty as defined in this review.

The two-year FINGER trial combined diet, exercise, cognitive training, and vascular-risk monitoring and produced a modest cognitive advantage among 1260 at-risk older adults [29]. A subsequent post hoc analysis, in which frailty was not the original primary outcome, reported reduced phenotypic pre-frailty or frailty through seven years, with attenuation by 11 years [30]. These findings cannot establish an effect on psychological frailty or identify the active component of the package.

More proximal evidence comes from the MIND-STEP secondary analysis: 156 nursing-home residents with mild cognitive impairment were randomized to 12 weeks of multicomponent exercise or usual care; psychological frailty was measured as a domain at baseline and at 6, 12, and 24 weeks [31]. At 12 weeks, the adjusted between-group risk difference was −13.87 percentage points (95% CI −25.04 to −2.69; *p* = 0.015). Enrollment was based on cognitive impairment, and the secondary result does not validate an affective–adaptive psychological-frailty definition. A 2025 dual-task exercise trial reported a TFI total-score difference but did not provide a distinct psychological-domain result; it is therefore recorded as an exclusion from the construct-specific result map (Supplementary Table S4).

Exercise prescription must be co-designed around safety, baseline capacity, pain, fall risk, transport, and preference. A minimal plan that is repeated is more valuable than an ideal plan that is abandoned. Pairing graded activity with behavioral activation, a walking companion, or a meaningful destination may improve adherence because the activity carries emotional and social value. Where nutrition, oral health, or medication-related dizziness limits participation, those enabling conditions require attention first.

### 5.5. Social Connection and Social Prescribing

Loneliness and social isolation are related but distinct: loneliness is a painful subjective discrepancy, whereas isolation concerns objective contact or network structure. Interventions must match the mechanism. In a 2022 systematic review and meta-analysis of 70 studies involving 8259 older adults, a range of interventions was associated with reduced loneliness or isolation, but heterogeneity was substantial [32]. A later systematic review similarly found that no single intervention type was uniformly effective and emphasized limits in study quality and comparability [33].

Social prescribing can connect people to community, cultural, creative, welfare, exercise, or volunteering resources. Meta-review evidence suggests that group activities, support with education, recreation, and communication technologies can help, but effectiveness depends on real-world implementation [34]. Reviews focused on older adults describe weak or heterogeneous causal evidence and recurring access barriers [35]. A referral therefore does not constitute an intervention unless it includes relational matching, practical access, and follow-up. Transport, cost, hearing, language, fear of unfamiliar groups, caregiving responsibilities, and whether the activity feels meaningful can determine success.

D-SCOPE provides a closer but cautionary comparison [36,37]. In its protocol, participants could qualify through psychological frailty (CFAI-Plus psychological score ≥20), another frailty domain, or subjective frailty; recruitment was not confined to the psychological domain. In the later evaluation, 540 participants were randomized (271 intervention; 269 control). At six months, the authors reported no significant within-group change in psychological frailty in either arm; they did not establish an intervention benefit for that domain by a reported adjusted between-arm contrast. Attrition in the control and intervention groups was 32% and 26%, respectively, and 77 intervention participants (28%) were not exposed to the program. Positive reports of useful visits and satisfaction concern implementation, not demonstrated psychological-frailty efficacy.

### 5.6. Sleep and Reversible Clinical Drivers

Sleep is a high-leverage target because insomnia worsens emotion regulation, fatigue, cognitive efficiency, pain, and activity. In an RCT of 291 older adults with insomnia, CBT for insomnia reduced the incidence or recurrence of major depression over 36 months compared with sleep education (hazard ratio 0.51) [38]. This does not imply that every sleep complaint requires a full protocol. Stimulus control, regular wake time, light exposure, activity scheduling, and medication review may be sufficient first steps, with escalation when insomnia persists.

Other reversible drivers are best viewed as enabling interventions. Hearing or vision support may reduce cognitive load and make psychotherapy or group participation possible. Pain treatment can restore sleep and movement. Nutrition and oral-health assessment can address fatigue and weight loss. Medication review can identify anticholinergic burden, sedative effects, dizziness, or interactions. The 2024 Lancet Commission on dementia prevention reinforced the relevance of depression, social isolation, hearing loss, vision loss, inactivity, and vascular risk across the life course [39]. Yet correcting a driver is not the same as treating all psychological distress; the point is to remove obstacles to adaptation and participation.

### 5.7. Digital and Gerontechnology-Supported Care

Digital delivery can extend psychotherapy, coaching, monitoring, cognitive training, and social contact, but population coverage must be stated accurately. Hansen et al. included mixed-age trials, approximately one-third of which focused on younger participants; their review therefore cannot be treated as older-adult-specific evidence [40]. A 2026 meta-analysis of 17 studies involving 2423 older participants reported a small-to-moderate reduction in loneliness (SMD −0.39), with high heterogeneity and uncertain durability [41]. Neither review tested psychological frailty.

Technology is a channel, not a therapeutic mechanism. A technically functional platform can still fail through poor usability, inaccessible text or audio, low trust, data costs, limited broadband, weak privacy protections, or lack of onboarding. Human facilitation frequently determines whether a digital tool is used long enough to matter. Hybrid care should preserve non-digital routes; otherwise, an intervention intended to reduce inequity may deepen it.

A further multidomain example is a Valencia trial that randomized 242 community-dwelling adults aged over 65 years to a 12-month individualized program or usual care [42]. The TFI psychological domain was measured, but the report describes only a small decrease in that domain and no statistically significant broad improvement. The trial was not restricted to participants enrolled on the basis of psychological frailty and its domain should not be read as a validated measure of the proposed construct.

### 5.8. What the Evidence Does—and Does Not—Support

The intervention literature contains distinct levels of proximity. D-SCOPE allowed psychological frailty as one of several eligibility domains, while Freitag and Schmidt, MIND-STEP, and Valencia explicitly measured frailty or a psychological domain [24,31,36,37,42]. These studies have limited and non-equivalent construct correspondence; D-SCOPE found no significant within-arm change in psychological frailty, and MIND-STEP showed a favorable secondary domain result. Adjacent symptom, sleep, exercise, and social studies support only their measured outcomes [17,18,19,20,21,22,23,25,26,27,28,29,30,32,33,34,35,38,39,40,41,43]. No identified trial exclusively enrolled a cohort defined by a validated affective–adaptive psychological-frailty construct. Heterogeneous outcome definitions, incomplete between-arm contrasts, and uneven reporting of burdens and harms prevent a pooled or causal claim about psychological reserve.

Integration should mean coordinated personalization, not assumed synergy. Adding components can increase fatigue, travel, cost, conflicting advice, and nonadherence. The relevant research question is whether a provisional tailoring strategy can identify an initial minimum-burden plan that improves a person-valued outcome and specifies when to continue, adapt, or stop. The present review does not establish such a strategy’s effectiveness. Table 2 summarizes the intervention evidence and its directness.

## 6. The RESERVE-7 Heuristic

**Figure 1:**
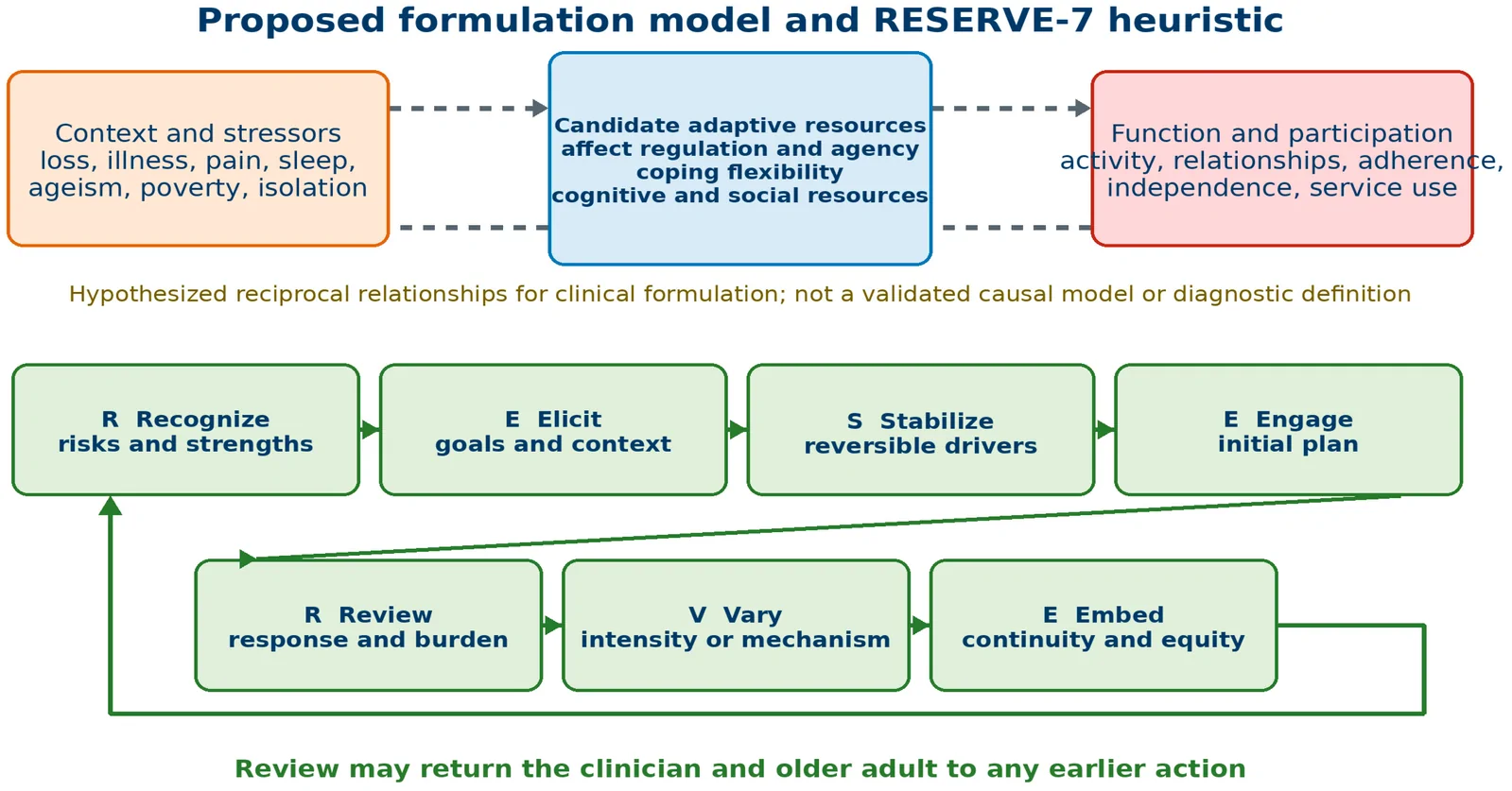
Proposed formulation model and RESERVE-7 heuristic. Dashed connections in the upper panel represent hypothesized relationships among context, adaptive resources, and function; they do not establish causal effects. Solid green arrows indicate the proposed sequence of RESERVE-7 actions, and the return loop indicates reassessment of earlier actions. RESERVE-7 displays all seven actions and a return loop; it is an author-derived mnemonic, not a measure of psychological reserve or a validated clinical pathway.

The reviewed literature informed an author-derived seven-action mnemonic, RESERVE-7 (Figure 1 and Table 3). The word is used only as an initialism for the seven actions; it is not a definition, score, or synonym for psychological reserve. Development was deductive and iterative: the authors mapped recurring concerns about strengths, goals, reversible factors, coordinated care, monitoring, treatment workload, continuity, and equity to a provisional sequence and refined it by discussion. Development did not involve a Delphi process, a formal consensus method, an independent panel, or co-design with patients and caregivers. RESERVE-7 should therefore be described as a proposed heuristic for research and structured discussion, not as a clinical pathway or treatment standard.

RESERVE-7 overlaps with established models. ICOPE begins with person-centered assessment of intrinsic capacity and social-care needs [2]; comprehensive geriatric assessment coordinates multidomain evaluation and management [44]; collaborative and stepped care contributes systematic follow-up and treatment adjustment [20]; and minimally disruptive medicine asks whether care workload exceeds a person’s capacity [16]. The proposed addition is an explicit, testable link between a hypothesized feedback loop, one primary intervention mechanism, an enabling component when needed, and repeated assessment of burden. Whether that addition improves decisions or outcomes is unknown.

### 6.1. Selecting an Initial Minimum-Burden Plan

The heuristic proposes choosing an initial mechanism by asking which feedback loop is most closely associated with loss of function and which change is feasible and valued. Behavioral activation might be considered when avoidance maintains inactivity; CBT for insomnia (CBT-I) might be considered when persistent insomnia contributes to fatigue; and hearing support might be considered when communication difficulty contributes to withdrawal. These are illustrative formulations, not prescriptive rules, and each requires diagnostic assessment, consent, and appropriate clinical guidance.

An initial plan may be limited to one primary intervention plus one enabling component when necessary. A prespecified review can examine goal progress, symptoms, function, adverse effects, adherence, feasibility, and workload. Improvement may support continuation; partial improvement may justify adaptation; and no improvement should prompt reassessment of diagnosis, delivery, access, goal relevance, harms, and competing mechanisms. Deterioration or acute risk requires established escalation procedures. These adaptation considerations have not been prospectively validated.

### 6.2. Shared Decisions and Treatment Burden

Shared decision making is especially important when multimorbidity or reduced capacity increases treatment workload. A review identified time, information, power, cognitive demands, caregiver dynamics, and organizational constraints as relevant barriers or facilitators [15]. Choices should be explained in accessible language, unnecessary simultaneous decisions should be avoided, and preferences should be revisited as circumstances change. A practical question is whether the expected benefit justifies the work the intervention requires from this person [15,16].

## 7. Culture, Equity, and Implementation

### 7.1. Cultural Responsiveness Beyond Translation

Cultural adaptation should address more than translated materials. Explanatory models of distress, family roles, spirituality, stigma, communication norms, migration history, collective identity, and expectations of professional help can alter the meaning and acceptability of every module. A systematic review of cultural-adaptation frameworks identified substantial variation but recurring attention to language, persons, metaphors, content, concepts, goals, methods, and context [45]. Adaptation should preserve an intervention’s plausible active mechanism while changing its delivery and framing through partnership with the communities concerned.

The concept of resilience deserves particular care. Praising resilience can inadvertently transfer responsibility from institutions to people who face poverty, racism, unsafe housing, inaccessible transport, or inadequate care. Reserve is partly produced by relationships and environments. A clinically responsible formulation therefore distinguishes skills that can be strengthened from resources that must be supplied and structural harms that must be addressed.

### 7.2. Social Infrastructure and Relational Continuity

Social connection is a public-health determinant, not a lifestyle luxury. Social isolation and loneliness are associated with mortality risk [46], and the WHO has called for coordinated action that includes policy, community infrastructure, and health services [47]. The National Academies similarly emphasized that health systems can identify risk and connect people to resources but cannot solve social isolation through screening alone [48]. Integration therefore requires durable relationships with community organizations, accessible transport, benefits and housing advice, culturally safe programs, and feedback to referrers.

Continuity is itself therapeutic. Repeated handoffs force an older adult to retell losses, renegotiate trust, and navigate conflicting plans. A named coordinator, shared formulation, and clear relapse pathway may preserve more reserve than an additional stand-alone session. Caregivers can support cueing, transport, home practice, and observation, but their own burden, autonomy, and relationship with the older adult must be assessed. Involvement should never be automatic.

### 7.3. Workforce and Service Design

Implementation requires role clarity. Primary-care teams can screen, stabilize common drivers, and coordinate; geropsychologists can provide formulation, adapted therapy, neuropsychological insight, and supervision; geriatricians and pharmacists can address multimorbidity and medication burden; physiotherapists and exercise professionals can grade activity; occupational therapists can translate goals into routines and environments; social workers and link workers can address resources and participation; and community partners can sustain belonging and purpose. Integration is achieved through shared decisions and feedback, not merely co-location.

Services should track reach as well as response. Uptake, early dropout, time and travel burden, accessibility accommodations, digital exclusion, caregiver strain, and differential outcomes by age, sex, ethnicity, language, socioeconomic position, rurality, disability, and cognitive or sensory status are essential implementation outcomes. Without these data, an apparently effective program may benefit only those already best positioned to use it.

## 8. Research Agenda

The field first needs conceptual discipline. Direct trials cannot accumulate coherently while eligibility depends on incompatible definitions. Consensus work should test whether an affective–adaptive construct predicts outcomes beyond depression, cognition, physical frailty, and social adversity, and whether it changes clinical decisions. Qualitative work should include people who accept, reinterpret, or reject frailty language. Measures must demonstrate content validity, responsiveness, interpretability, and cross-cultural invariance—not simply internal consistency.

Intervention research should then move beyond undifferentiated packages. Factorial, sequential multiple assignment randomized, and micro-randomized designs can test components and decision rules. Pragmatic cluster trials can evaluate implementation across primary care and community systems. Core outcomes should include person-valued goal attainment, participation, function, coping or self-efficacy, symptoms, quality of life, treatment burden, adverse events, caregiver outcomes, and health-service use. Follow-up should be long enough to distinguish transient activation from durable reserve.

Mechanism testing is essential. Mediation analyses can examine whether sleep, mastery, activity, social connection, or perceived control precedes functional benefit. Just as importantly, qualitative process evaluation can identify why a nominally identical module feels enabling to one person and burdensome to another. Realist approaches are appropriate where the causal question is how context activates or suppresses an intervention mechanism; narrative and qualitative evidence should not be placed below experimental evidence when the question concerns meaning, acceptability, or implementation [49]. Table 4 summarizes the priority research questions, proposed study designs, and outcomes.

## 9. Strengths and Limitations

This review integrates construct-specific and adjacent intervention evidence while separating enrollment, measured outcome, and conceptual concordance. The focused reconciliation corrected an earlier omission of five pertinent publications and the overbroad claim that all interventions were indirect. The original 114-record core yield remains historical and is not an exhaustive intervention census. Our working definition and RESERVE-7 remain unvalidated.

This critical narrative review was not preregistered and did not use exhaustive multidisciplinary database coverage, duplicate independent screening, a systematic review-management workflow, or formal risk-of-bias assessment. The construct-specific reconciliation was focused rather than exhaustive; further relevant trials may be missed, particularly when psychological-domain findings appear only in full text or supplements. We did not recover or independently recompute unreported effects. Positive within-arm results cannot substitute for between-arm tests, and a secondary domain result can be vulnerable to multiplicity. The English-language restriction, heterogeneous measures, attrition, selective reporting, and overlap among reviews further limit inference. Our definition and RESERVE-7 have not undergone consensus, construct validation, co-design, or prospective testing.

## 10. Conclusions

Psychological frailty is a contested construct, not a characteristic of older adulthood. It may prove useful only if it adds information beyond depression, cognition, physical frailty, social adversity, resilience, and intrinsic capacity and if its language does not stigmatize people or conceal structural causes.

Current evidence does not establish those conditions.

Evidence includes a small number of studies that explicitly assess psychological frailty or use it as one possible eligibility domain, alongside a broader but less direct literature on depression, insomnia, physical and cognitive frailty, loneliness, and access barriers. The construct-specific results are mixed and operational definitions differ. They cannot establish that interventions restore a distinct psychological reserve. RESERVE-7 remains a research heuristic requiring co-design, validation, and prospective testing before routine use.

## Figures and Tables

**Table 1:** Construct boundaries relevant to integrative geropsychology.

Construct	Core Question	Typical Indicators	Clinical Implication
Psychological frailty (provisional working definition)	Can the person regulate affect, sustain agency, and mobilize resources under stress?	Reduced coping flexibility, helplessness, withdrawal, low perceived control; context-dependent fluctuation	Formulate drivers and feedback loops; avoid treating it as a diagnosis or identity
Depressive disorder	Is there a syndromal disturbance of mood, interest, cognition, or biological function?	Persistent low mood/anhedonia, guilt, sleep/appetite change, suicidality	Assess diagnostic criteria, severity, risk, and evidence-based treatment
Cognitive frailty	Do physical frailty and cognitive impairment coexist without established dementia?	Physical frailty plus objective cognitive impairment	Clarify reversible causes, cognitive status, safety, and multimodal prevention
Social frailty	Are social roles, relationships, participation, or practical resources threatened?	Loneliness, isolation, reduced participation, loss of role or support	Treat barriers and social conditions; do not locate deprivation solely within the person
Resilience	How well does the person adapt despite adversity?	Recovery, flexibility, meaning, self-efficacy, resource use	Identify and strengthen existing assets; recognize that resilience is relational and contextual
Psychological reserve (author-defined formulation device)	Which affective, agentic, cognitive, social, and environmental resources are accessible under stress?	No validated indicator set; candidate measures include coping flexibility, self-efficacy, perceived control, and resource access	Do not treat as a diagnosis, latent trait, or outcome until operationalized and validated; distinguish it from resilience, cognitive reserve, and intrinsic capacity [2,7,8,12]

**Table 2:** Intervention evidence by module and construct-specific study. P denotes population directness; O, outcome directness; C, concordance with the proposed affective–adaptive construct. See Supplementary Table S4 for study-level reporting fields.

Module and Sources	Population and Directness	Evidence Signal	Limitations, Harms, and Workload
Psychological therapies and collaborative care [17,18,19,20]	Late-life subthreshold depression, major depression, anxiety, or primary-care depression. Adjacent; no enrollment based on psychological frailty.	13 RCTs, *n* = 2079: depressive symptoms SMD −0.58 post-intervention and −0.31 at follow-up [17]. IMPACT *n* = 1801 supports collaborative depression care [20].	Heterogeneous formats and comparators; adaptations, adherence, medication effects, and burden require individual assessment.
Mindfulness and mind–body approaches [21,22,23,43]	Older adults with depression, anxiety, stress, or mixed health status. Adjacent.	Benefits reported for selected mood, anxiety, stress, quality-of-life, sleep, and resilience outcomes; cognition inconsistent.	Practice time, pain, trauma-related distress, cognitive load, cultural fit, attrition, and adverse-event reporting vary.
Life review, reminiscence, and meaning [25,26]	Older adults with late-life depression or without marked cognitive impairment. Adjacent.	Generally favorable depressive-symptom and well-being findings, with heterogeneous formats, settings, and comparators.	Possible distress when traumatic memories emerge; sensory and cognitive accessibility and facilitator skill matter.
Exercise and multidomain lifestyle [27,28,29,30]	Cognitive or physical frailty and dementia-risk samples. Adjacent.	11 trials, *n* = 2222, reported selected cognitive, physical, frailty, and depressive benefits [27]. FINGER frailty result was post hoc [30].	Falls, pain, access, adherence, and maintenance matter. Multicomponent packages obscure active ingredients; no psychological reserve outcome.
Social connection and social prescribing [32,33,34,35]	Loneliness or isolation in older adults; social-prescribing reviews. Adjacent.	A review of 70 studies (*n* = 8259) reported reductions in loneliness or isolation, but heterogeneity and study-quality concerns were substantial [32].	Referral alone may fail. Relational fit, transport, cost, hearing, language, acceptability, and follow-up shape uptake and burden.
Sleep and enabling clinical care [38,39]	Older adults with insomnia; dementia-risk literature. Adjacent.	CBT-I trial (*n* = 291): incident or recurrent major depression HR 0.51 over 36 months [38].	CBT-I workload and adherence vary. Sensory, pain, nutrition, and medication interventions enable participation but do not establish treatment of psychological frailty.
Digital and hybrid delivery [40,41]	Mixed-age digital social-connection evidence [40] and older-adult loneliness studies [41]. Adjacent.	17 studies, *n* = 2423: loneliness SMD −0.39, with high heterogeneity and uncertain durability [41].	Digital exclusion, privacy, usability, onboarding, support, dropout, data cost, and short-lived engagement require explicit reporting.
Freitag and Schmidt [24]	Community older adults, *n* = 198; P no; O TFI total (psychological subscale effect not separately reported); C uncertain.	39.9% frail at baseline; small improvements in frailty and mental health in selected narrative/diary arms; 3-month follow-up.	Reported: no physical-health effect. Other reporting fields and inference: Supplementary Table S4.
D-SCOPE [36,37]	Belgian community adults ≥60; *n* = 540 randomized; P possible via CFAI-Plus psychological domain among other routes; O yes; C partial.	At six months no significant within-arm psychological-domain change; between-arm domain effect not established in the report.	Reported: attrition 32% control, 26% intervention; non-exposure 28% intervention; acceptability assessed.
MIND-STEP [31]	Chinese nursing-home adults with MCI; *n* = 156; P no; O yes; C partial/uncertain.	12-week psychological-frailty RD −13.87 percentage points (95% CI −25.04 to −2.69); assessed again at 24 weeks.	Secondary analysis; long-term durability, workload and complete safety reporting not established from accessible abstract.
Valencia [42]	Spanish community adults > 65 with frailty; *n* = 242; P no psychological-specific route; O TFI psychological domain; C partial.	12-month program; small psychological-domain reduction, without a clearly established significant broad frailty benefit.	Reported study limitations and missing implementation fields: Supplementary Table S4.

**Table 3:** RESERVE-7 as a proposed, unvalidated heuristic for structured clinical reasoning.

Action	Clinical Task	Minimum Documentation	Provisional Consideration
R: Recognize risks and strengths	Screen mood, anxiety, cognition, sleep, pain, mobility, nutrition, sensory function, loneliness, medicines, safety, and retained assets	Key risks; one or two strengths; urgent exclusions	Use established urgent assessment and escalation procedures before applying the heuristic
E: Elicit goals, culture, and meaning	Ask what the person wants to regain, protect, or contribute; clarify language, beliefs, identity, and acceptable support	One person-valued goal in the person’s words	Proceed only with relevant and consented options
S: Stabilize reversible drivers	Address delirium risk, sleep, pain, sensory loss, nutrition, medication burden, transport, and safeguarding	Driver, responsible clinician/service, time frame	Consider the factor most likely to prevent engagement; coordinate with appropriate clinicians
E: Engage an initial minimum-burden plan	Select one hypothesized primary mechanism and, if needed, one enabling component	Intervention, dose, access adaptation, expected outcome, and uncertainty	Avoid adding components without a stated rationale; effectiveness of this strategy is untested
R: Review response and burden	Reassess goal progress, symptoms, function/participation, coping, adherence, adverse effects, and effort	Outcome trend plus burden/feasibility	Continue, adapt, or stop through shared decisions; no validated threshold exists
V: Vary intensity or mechanism	Step up, step down, switch mechanism, or simplify delivery	Reason for adaptation and next review	Nonresponse should prompt reassessment before adding treatment
E: Embed continuity, caregivers, and equity	Plan maintenance, relapse signals, handover, consented supporter roles, and non-digital access	Named follow-up route and contingency plan	Agree on a feasible follow-up and re-entry plan when ongoing vulnerability is anticipated

**Table 4:** Priority research questions for psychological frailty and integrative intervention.

Gap	Recommended Design	Decisive Outcomes
Construct validity	Consensus study followed by prospective validation against adjacent constructs	Incremental prediction; responsiveness; cross-cultural invariance; clinical utility
Component selection	Factorial or sequential multiple assignment randomized trial	Active components; tailoring variables; burden-adjusted benefit
Mechanisms	Longitudinal mediation plus embedded qualitative process evaluation	Temporal change in sleep, agency, activity, connection, and function
Real-world effectiveness	Pragmatic cluster trial across health and community settings	Goal attainment, participation, function, service use, reach, fidelity, cost
Equity and accessibility	Oversampled and co-designed studies including oldest-old, sensory/cognitive disability, rural and minoritized groups	Differential uptake, dropout, harms, accessibility, and outcomes
Durability	Maintenance and deprescribing studies with at least 12-month follow-up	Sustained reserve, relapse, ongoing burden, ability to step down safely

## Data Availability

No new data were created or analyzed in this study. The sources synthesized are cited in the article.

## Supplementary Materials

The following supporting information can be downloaded at: https://www.mdpi.com/article/10.3390/healthcare14203423/s1, Table S1: PubMed Search Strategy; Table S2: Eligibility and Evidence Classification; Table S3: SANRA Self Audit; Table S4: Construct Specific Intervention Reconciliation. Reference [50] is cited in the Supplementary Materials.
