# Supplementary material for: Psychological Frailty in Later Life: A Critical Narrative Review of Integrative Interventions and the Proposed RESERVE-7 Heuristic
Source: Healthcare (Basel). 2026 Oct 13;14(20):3423. doi: 10.3390/healthcare14203423 (PMC13704219; doi:10.3390/healthcare14203423)
Supplement: Supplementary file 1 [file healthcare-14-03423-s001.zip › healthcare-4563210-supplementary.pdf]

Supplementary File S1 Search Strategy and SANRA Self Audit

This supplement documents MEDLINE via PubMed searches conducted through 29 August 2026 and a subsequent focused reconciliation of construct-specific intervention reports prompted by peer review. The original core query returned 114 records; this number was not recomputed as an exhaustive intervention count. Forty-nine sources inform the revised manuscript (44 previously cited plus five added construct-specific publications). Targeted searches and citation chasing had no deduplicated screening register. Supplementary Table S4 records the focused inclusion and exclusion decisions. “Not reported” means not located in the consulted report, not evidence that the study did not collect the item.

Supplementary Table S1 PubMed Search Strategy

ID	Purpose	Exact PubMed string
S1	Core construct	(("psychological frailty"[Title/Abstract] OR "affective frailty"[Title/Abstract] OR "mental frailty"[Title/Abstract] OR "psychological reserve"[Title/Abstract]) AND ("Aged"[Mesh] OR older adult*[Title/Abstract] OR elderly[Title/Abstract] OR ageing[Title/Abstract] OR aging[Title/Abstract])) AND ("2015/01/01"[Date - Publication] : "2026/08/29"[Date - Publication]) AND English[Language]
S2	Psychological therapies	("Aged"[Mesh] OR older adult*[Title/Abstract] OR elderly[Title/Abstract] OR late-life[Title/Abstract] OR geriatric*[Title/Abstract]) AND (depress*[Title/Abstract] OR anxiety[Title/Abstract] OR psychological frailty[Title/Abstract]) AND (psychotherap*[Title/Abstract] OR cognitive behavioral therapy[Title/Abstract] OR cognitive behavioural therapy[Title/Abstract] OR problem-solving therap*[Title/Abstract] OR behavioral activation[Title/Abstract] OR behavioural activation[Title/Abstract] OR collaborative care[Title/Abstract] OR stepped care[Title/Abstract]) AND ("2015/01/01"[Date - Publication] : "2026/08/29"[Date - Publication]) AND English[Language]
S3	Mindfulness and mind-body	("Aged"[Mesh] OR older adult*[Title/Abstract] OR elderly[Title/Abstract] OR late-life[Title/Abstract] OR geriatric*[Title/Abstract]) AND (mindfulness[Title/Abstract] OR mind-body[Title/Abstract] OR meditation[Title/Abstract]) AND (depress*[Title/Abstract] OR anxiety[Title/Abstract] OR stress[Title/Abstract] OR resilience[Title/Abstract] OR sleep[Title/Abstract]) AND ("2015/01/01"[Date - Publication] : "2026/08/29"[Date - Publication]) AND English[Language]
S4	Life review and meaning	("Aged"[Mesh] OR older adult*[Title/Abstract] OR elderly[Title/Abstract] OR late-life[Title/Abstract] OR geriatric*[Title/Abstract]) AND (reminiscence[Title/Abstract] OR life review[Title/Abstract] OR meaning-centered[Title/Abstract] OR meaning-centred[Title/Abstract]) AND (depress*[Title/Abstract] OR wellbeing[Title/Abstract] OR well-being[Title/Abstract] OR quality of life[Title/Abstract]) AND ("2015/01/01"[Date -

ID	Purpose	Exact PubMed string
		Publication] : "2026/08/29"[Date - Publication]) AND English[Language]
S5	Exercise and multidomain lifestyle	("Aged"[Mesh] OR older adult*[Title/Abstract] OR elderly[Title/Abstract] OR late-life[Title/Abstract] OR geriatric*[Title/Abstract]) AND (frailty[Title/Abstract] OR cognitive frailty[Title/Abstract]) AND (exercise[Title/Abstract] OR physical activity[Title/Abstract] OR multidomain[Title/Abstract] OR multicomponent[Title/Abstract] OR lifestyle[Title/Abstract]) AND ("2015/01/01"[Date - Publication] : "2026/08/29"[Date - Publication]) AND English[Language]
S6	Social connection	("Aged"[Mesh] OR older adult*[Title/Abstract] OR elderly[Title/Abstract] OR late-life[Title/Abstract] OR geriatric*[Title/Abstract]) AND (loneliness[Title/Abstract] OR social isolation[Title/Abstract] OR social connection[Title/Abstract] OR social prescribing[Title/Abstract]) AND (intervention*[Title/Abstract] OR trial[Title/Abstract] OR review[Title/Abstract]) AND ("2015/01/01"[Date - Publication] : "2026/08/29"[Date - Publication]) AND English[Language]
S7	Sleep and enabling factors	("Aged"[Mesh] OR older adult*[Title/Abstract] OR elderly[Title/Abstract] OR late-life[Title/Abstract] OR geriatric*[Title/Abstract]) AND (insomnia[Title/Abstract] OR sleep[Title/Abstract] OR hearing loss[Title/Abstract] OR vision loss[Title/Abstract] OR pain[Title/Abstract] OR medication review[Title/Abstract]) AND (intervention*[Title/Abstract] OR therapy[Title/Abstract] OR trial[Title/Abstract] OR review[Title/Abstract]) AND ("2015/01/01"[Date - Publication] : "2026/08/29"[Date - Publication]) AND English[Language]
S8	Digital or hybrid care	("Aged"[Mesh] OR older adult*[Title/Abstract] OR elderly[Title/Abstract] OR late-life[Title/Abstract] OR geriatric*[Title/Abstract]) AND (digital health[Title/Abstract] OR telehealth[Title/Abstract] OR gerontechnology[Title/Abstract] OR internet-based[Title/Abstract] OR mobile health[Title/Abstract]) AND (psychotherap*[Title/Abstract] OR loneliness[Title/Abstract] OR social connection[Title/Abstract] OR mental health[Title/Abstract]) AND ("2015/01/01"[Date - Publication] : "2026/08/29"[Date - Publication]) AND English[Language]

Supplementary Table S2 Eligibility and Evidence Classification

Item	Operational approach
Population	Mean or median age at least 60 years, at least 80% aged 60 years or older, or an extractable subgroup meeting the threshold.
Three directness dimensions	Population: exclusive psychological-frailty criterion, one possible psychological-domain route, or no such criterion. Outcome: distinct psychological-frailty domain reported, only

Item	Operational approach
	multidomain total reported, or neither. Construct concordance: match to the proposed affective–adaptive definition is assessed separately and never inferred merely from the term frailty.
Adjacent evidence	Other outcomes and populations are adjacent to the review question even if related to coping, depression, cognitive frailty, or physical frailty. A multidomain frailty total without psychological-domain analysis is not counted as a psychological-frailty effect.
Interventions	Psychological, behavioral, physical, social, existential, digital, collaborative, or enabling care relevant to the review question.
Exclusions	Pharmacotherapy-only studies; evidence with no interpretable relevance to later-life care; dementia-specific findings without transferable implementation or accessibility lessons.
Selection	F.M.I. performed searches and initial selection; both authors reviewed the final source set and resolved uncertainties by discussion.
Synthesis	Report design, enrollment route, instrument, population, N, result, follow-up, and whether attrition, adherence, adverse events, acceptability, and workload are reported. Separate source-documented limitations from author-inferred cautions. No meta-analysis or formal risk-of-bias scoring.

Supplementary Table S3 SANRA Self Audit

SANRA domain	Revision evidence	Location
Importance	Clinical relevance and risk of construct overlap are explained without framing aging as pathology.	Introduction
Aims	Three explicit aims distinguish conceptual clarification, adjacent intervention evidence, and heuristic development.	End of Introduction
Literature search	Database, dates, exact Boolean strings, core record count, eligibility thresholds, selection roles, and limitations are reported.	Methods and Supplementary Tables S1-S2
Referencing	Foundational, recent, intervention, implementation, and competing-framework sources are cited; selected bibliographic errors were corrected.	Throughout and References
Scientific reasoning	Direct evidence, adjacent evidence, proposed mechanisms, and author-derived inference are separated.	Methods, Table 2, Sections 3-6
Endpoint data	Effect estimates and sample sizes are reported where available; heterogeneity, durability, and missing burden/harms reporting are noted.	Section 5 and Table 2

Supplementary Table S4 Construct Specific Intervention Reconciliation

Focused reconciliation of reports identified by reviewer feedback and targeted searches; no claim of exhaustive identification. P = population directness; O = outcome directness; C = concordance with the proposed affective–adaptive construct. Study-reported items and our inferences are kept separate.

Study and decision	Population and operational definition	Design N result and follow-up	Reporting of implementation fields	Source limitations and author inference
Freitag and Schmidt 2016 [24] • Include	Community-dwelling older adults; mean 75.1 y. TFI total frailty, which contains a psychological domain. P no psychological-specific enrollment; O frailty total, separate psychological effect not located; C uncertain.	Randomized narrative/diary conditions and control; N=198; 39.9% frail at baseline. Small frailty and mental-health benefits in oral, structured writing, and diary groups; no physical-health effect. Baseline, post, three months.	Attrition, adherence, adverse events, acceptability, workload: not reported in accessible abstract. Diary/writing task described, but quantitative workload not reported.	Source: small effects and short follow-up. Author inference: TFI total effects cannot be attributed to its psychological subscale.
D-SCOPE protocol 2018 [36] • Include as protocol	Belgian community adults ≥60; CFAI-Plus psychological ≥20 could qualify, as could another of four domains or subjective frailty. P one possible route; O planned psychological domain; C partial/uncertain.	Four-arm randomized protocol; planned N=900; baseline and six months. No efficacy result: protocol only.	Attrition and adherence: planned measurement. Adverse events, acceptability, participant workload: not reported as outcomes in consulted protocol.	Source: design only. Author inference: planned N must not be conflated with 540 randomized in subsequent evaluation.
Dierckx et al. 2022 D-SCOPE [37] • Include results	Belgian community adults; multidomain CFAI/CFAI-Plus psychological score. P psychological domain could qualify among alternatives; O yes; C partial/uncertain.	Randomized evaluation N=540 (271 intervention, 269 control); six months. Neither group showed significant within-group psychological-frailty change; adjusted between-arm domain estimate not provided in consulted report.	Attrition: 26% intervention, 32% control. Exposure: 77/271 (28%) intervention participants did not receive program. Acceptability: 83.9% found visits useful; 82.6% satisfied. Adverse events, participant workload: not reported.	Source: substantial non-exposure and dropout; usual care not systematically recorded; analysis describes paired within-arm tests. Author inference: satisfaction cannot establish comparative efficacy.
Hong et al. 2026 MIND-STEP [31] • Include	Chinese nursing-home adults ≥60 with MCI, no regular exercise. Psychological frailty measured as separate domain. P no; O yes; C uncertain.	Secondary analysis of randomized trial; N=156; exercise versus usual care for 12 weeks; assessments at 0, 6, 12, 24 weeks. At 12 weeks psychological-frailty RD −13.87% (95% CI −25.04 to −2.69; p=.015).	Attrition, adherence, adverse events, acceptability, participant workload: not reported in consulted article abstract; full trial materials should be checked before asserting absence.	Source: secondary analysis; follow-up to 24 weeks. Author inference: interpretation limited by secondary outcome and uncertain equivalence of construct.
Fernández-Salido et al. 2024 [42] • Include	Spanish community adults >65 with frailty; TFI psychological subscale assessed. P no psychological-specific enrollment; O yes; C partial.	Randomized pre/post comparison N=242 (122 intervention, 120 control); personalized program over 12 months; small decrease in psychological domain, without demonstrated significant broad improvement.	Attrition, adherence, adverse events, acceptability, participant workload: not consistently available in consulted abstract and excerpts; verify in source for submission.	Source: conclusions do not support broad significant improvement. Author inference: cannot infer validated affective–adaptive benefit.
Lai et al. 2025 [50], doi:10.3389/fnagi.2025.1639245 • Exclude from psychological-domain result rows	Chinese adults ≥60 with cognitive frailty; TFI contains psychological items. P no; O only TFI total reported; C not assessable.	Randomized dual-task training versus education; N=72; 16 weeks. TFI total at endpoint 5.14±0.99 versus 7.36±1.07 (p<0.001); no separately reported psychological-domain effect.	Adherence: ≥80% prespecified; family help and app check-ins described. Other reporting not extracted for this excluded row.	Source: no follow-up beyond intervention; inconsistent session descriptions within article. Author inference: total TFI change is not evidence of a psychological-domain effect.
